# Abnormal levels of histone methylation in the retinas of diabetic rats are reversed by minocycline treatment

**DOI:** 10.1038/srep45103

**Published:** 2017-03-24

**Authors:** Wenjun Wang, Simone Sidoli, Wenquan Zhang, Qing Wang, Leilei Wang, Ole N. Jensen, Lin Guo, Xiaolu Zhao, Ling Zheng

**Affiliations:** 1 Hubei Key Laboratory of Cell Homeostasis, College of Life Sciences, Wuhan University, Wuhan, 430072, P.R. China; 2 Department of Biochemistry and Molecular Biology and VILLUM Center for Bioanalytical Sciences, University of Southern Denmark, DK-5230 Odense M, Denmark

## Abstract

In this study we quantified the alterations of retinal histone post-translational modifications (PTMs) in diabetic rats using a liquid chromatography - tandem mass spectrometry (LC-MS/MS) approach. Some diabetic rats were subsequently treated with minocycline, a tetracycline antibiotic, which has been shown to inhibit the diabetes-induced chronic inflammation in the retinas of rodents. We quantified 266 differentially modified histone peptides, including 48 out of 83 methylation marks with significantly different abundancein retinas of diabetic rats as compared to non-diabetic controls. About 67% of these marks had their relative abundance restored to non-diabetic levels after minocycline treatment. Mono- and di-methylation states of histone H4 lysine 20 (H4K20me1/me2), markers related to DNA damage response, were found to be up-regulated in the retinas of diabetic rats and restored to control levels upon minocycline treatment. DNA damage response biomarkers showed the same pattern once quantified by western blotting. Collectively, this study indicates that alteration of some histone methylation levels is associated with the development of diabetic retinopathy in rodents, and the beneficial effect of minocycline on the retinas of diabetic rodents is partially through its ability to normalize the altered histone methylation levels.

Diabetic retinopathy (DR)is one of the microvascular complications of diabetes and the leading cause for blindness among the working adults^1^. Histone proteinscan be differentially modified in the retinas of non-diabetic and diabetic rodents, and in cultured Müller cells and retinal endothelial cell sunder diabetes-like conditions^2^,^3^,^4^. Increased acetylation levels on histones promote transcription of inflammatory genes, which contribute to the pathogenesis of diabetic retinopathy^2^,^4^. Alteration of H3K4me1/me2 marks associated with down-regulation of the key anti-oxidative enzyme manganese superoxide dismutase(MnSOD) is found in the retinas of diabetic rats and endothelial cells cultured under the diabetic-like conditions^3^. All these studies suggest that epigenetic modifications, especially histone post-translational modifications (PTMs), play important roles in the development of diabetic retinopathy. However, no systematic study of histone PTMs in diabetic retinopathy is currently available.

Minocycline is a second-generation tetracycline. Besides its antimicrobial and anti-infection effects, minocyclinealso has a strong neuro-protective effect in cultured neuronal cells and animal models of neurodegenerative diseases^5^,^6^. Furthermore, minocycline has been demonstrated to have beneficial effects on diabetic retinopathy in rodent models. Krady *et al*. demonstrated that minocycline represses the expression of inflammatory factors, prevents the release of cytokines from the activated microglia, as well as reduces the caspase-3 dependent apoptosis in the retinas of diabetic rats^7^. Furthermore, minocycline significantly inhibits the diabetes-induced capillary degeneration in the retinas of diabetic mice by repressing the activity of caspase-1 and the expression of Il-β, two markers of inflammation^8^. The inhibitory effects of minocycline on inflammatory response are possible due to its ability to inhibit the diabetes- or hyperglycemia-induced histone hyperacetylation^2^,^4^. Glial activation, which is marked by increased glial fibrillary acidic protein (GFAP), is a phenotype in many ophthalmic diseases including diabetic retinopathy^9^,^10^. Minocycline has been found to inhibit glial activation, as well as neurodegeneration and inflammation in brain^11^. However, whether minocycline has effects on diabetes-induced glial activation in the retinas is not known.

Currently, the most effective methodology to systematically characterize histone PTMs is mass spectrometry(MS)-based proteomics^12^,^13^,^14^. Proteolytic protein digestion using trypsin or other proteases is the most widespread methodfor fast and accurate characterization of singlePTMs on core histones and histone variants^14^,^15^. Additionally, MS-based histone analysis can provide information for adjacent(co-existing) marks, assuming the two modified sites are present on the same peptide^16^,^17^. Recently, we reported a quantitative map of single and adjacent co-existing histone PTMs in rat retinas affected by ischemia and reperfusion (I/R) injury^18^. This study confirmed that histones can be extracted from retina and their PTMs can be accurately quantified. We thus decided to apply a similar workflow for the analysis of histone marks in the retinas of diabetic animal models.

In the present study, we performed an initial screening of histone proteins from the retinas of non-diabetic and diabetic rats with or without minocycline treatment using MS, followed by validation using western blotting. Our results demonstrate that histonePTMs, particularly histone methylations, have a different abundance in disease as compared to control, and that minocycline treatment contributes to revert most ofthe PTM levels to the non-diabetic state. Associated with increased DNA damage response, H4K20me1/me2 was found up-regulated in the retinas of diabetic rats. Interestingly, the same modification was also up-regulated upon retinal I/R injury, as characterized in our previous study^18^, suggesting that DNA damage response ininjured retina is a highly active and dynamic process. Minocycline treatment contributed to revert the levels of H4K20me1/me2, suggesting that DNA damage response was reduced. In summary, our results suggest that histone PTMs are involved in the etiology and/or pathology of diabetic retinopathy.

## Results

### Profiling histone PTMs in the retinas of experimental groups by MS analysis

**Figure 1:**
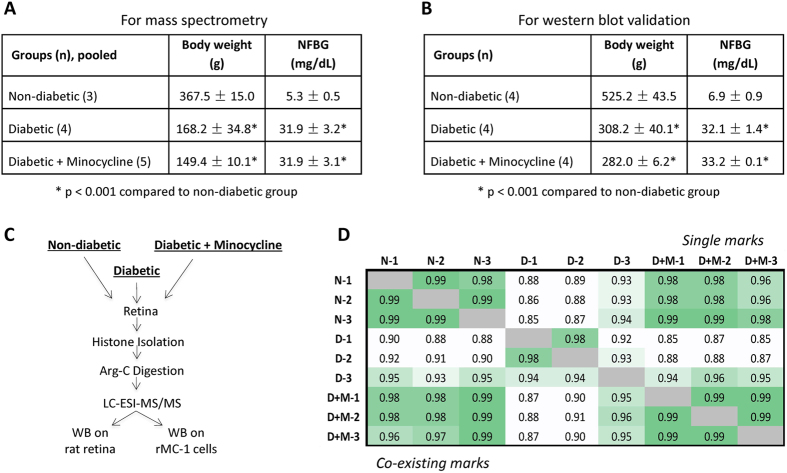
(**A**) Physical characterizes of animals used in the present study for MS analysis and (**B**) for western blot validation.(**C**) Workflow. Histone PTMs from rat retinas under health, diabetes and minocycline treatment were characterized by label-free quantitative proteomics in triplicates in this study. (**D**) Correlation between replicate analyses. R^2^ correlation values of the relative abundance of histone marks between replicates and between samples. N, D and D + M indicate control, diabetic and diabetic with minocycline samples, respectively. On the top right, correlation values for the relative abundance of single marks. On the bottom left, same for the co-existing marks. The average R^2^ for single and co-existing marks were 0.93 and 0.95, respectively, indicating a good correlation between replicates and samples.

In this study we profiled the alterations of retinal histone PTMs in the retinas of diabetic rats with or without treatment of minocycline. At first, we performed a quantitative analysis of histone PTMs using nano liquid chromatography coupled to MS. The physical characteristics of the experimental groups are shown in Fig. 1A for MS analysis and Fig. 1B for western blot validation. The streptozocin (STZ)-induced elevated blood glucose level and body weight loss was not affected by minocycline treatment. Typically, in type-1 diabetes the organism does not gain weight, or even loses, but the glucose content in the blood increases dramatically. This was confirmed by our observations of rat weight and blood glucose levels. The MS-based proteomics workflow is illustrated in Fig. 1C. Briefly, histones were digested using endoproteinase Arg-C and analyzed in three technical replicates by MS. To ensure sufficient sensitivity, we merged eye retina from multiple rats (i.e. 3 rats for non-diabetic, 4 for diabetic and 5 for diabetic + minocycline treatment). Quantification was performed as previously described^18^. In brief, each modified state was divided by the total histone abundance, estimated by summing the intensity of the modified + unmodified peptide, achieving a percentage that should approximately represent the stoichiometry of the PTMs. Since some peptides could contain multiple modified forms (e.g. histone H3 peptide aa 9–17 contains K9, K14 and R17 modification sites) we could establish co-existence frequencies for some of the PTMs. The relative abundances of quantified histone marks were compared to determine the degree of correlation between all the performed replicate analyses under three conditions (Fig. 1D). Cells were color coded according to correlation value intensity. Both correlation values of the single marks and co-existing marks achieved good R^2^ linear correlations between replicates and samples, highlighting the reproducibility of the experimental workflow.

A total of 266 peptides derived from histone H3, H4, H2A, H2B, H1 and non-canonical variants were quantified in a label-free mode using the Progenesis LC-MS software. 155 of the 266 identified peptides carried between one and four PTMs. A total of 135 distinct histone marks were characterized, distributed as follows: 43 on histone H3, 19 on H4, 37 on H2A, 22 on H2B and 14 on H1 respectively (PTMs information for all histones and their variants is included in Table 1 and Supplementary Tables 1–4). The most frequent PTMs detected were acetylation and methylations (mono-, di- and tri-methylation) on lysine residues. Retinal histones were decorated with all forms of methylations including mono-methylation (30.4%), di-methylation (22.2%) and tri-methylation (8.9%) as well as acetylation (38.5%) (Supplementary Fig. 1).

**Figure 2:**
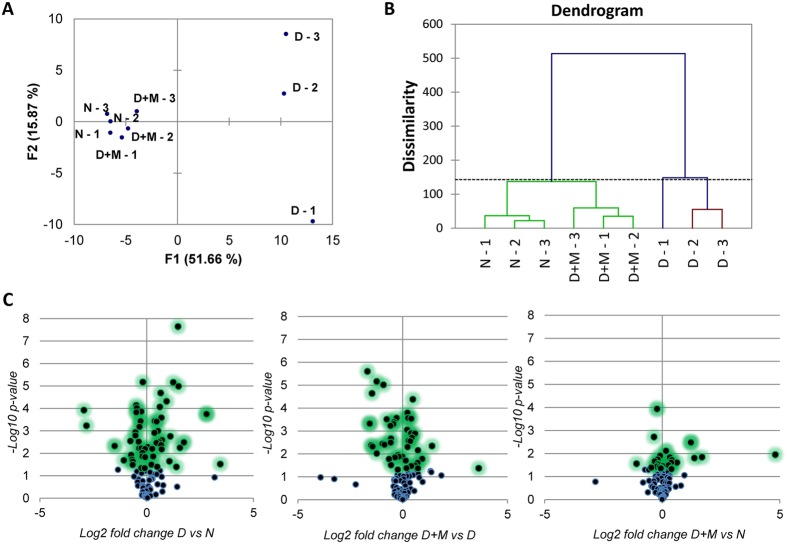
(**A**) Volcano plots representing the fold change (x-axis) and the significance (y-axis) for the single histone marks. The statistical difference is calculated using the t-test. Most significant changes are higher in the graph (in green). (**B**) Principal component analysis. (**C**) Hierarchical clustering.

**Figure 3:**
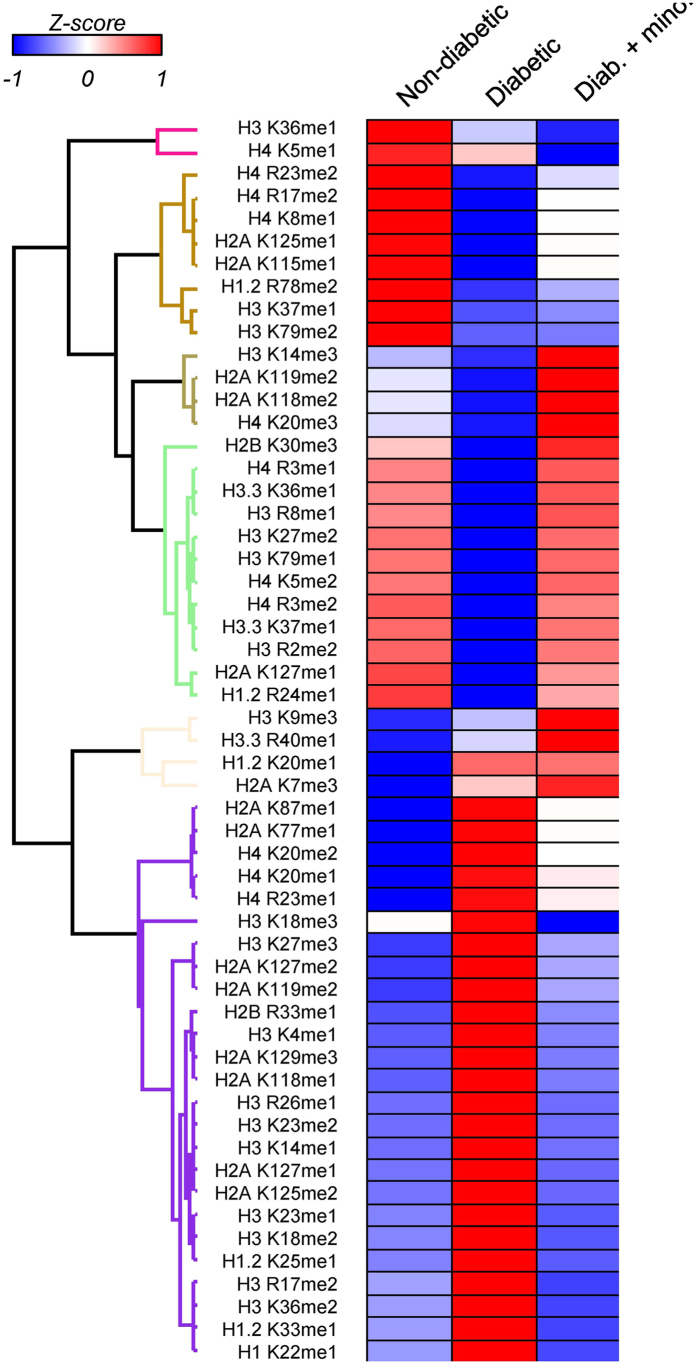
Heatmap of the major methylation marks found to be regulated across the three conditions.

Regardless of the similarity between all runs, we could clearly observe higher similarities between non-diabetic and minocycline treated retinas as opposed to the untreated diabetic state (Fig. 2A and B). We applied two types of clustering, namely principal component analysis (PCA, Fig. 2A) and agglomerative hierarchical clustering (AHC, Fig. 2B); both confirmed that replicates had excellent reproducibility, and that the diabetic rats without treatment differed from the other two states. Furthermore, in order to include statistical variance in the representation of our dataset we plotted fold changes and statistical *p*-value stogether using volcano plots. The comparison of non-diabetic vs minocycline treated diabetic showed the lowest numbers of statistically different marks (Fig. 2C). Of the 135 specific histone marks characterized in the present study, a total of 67 PTMs (including 22 in H3, 13 in H4, 18 in H2A, 6 in H2B and 8 in H1) showed significant up/down regulation in the retinas of diabetic rats when compared to non-diabetic rats(summarized in Table 1). 51marks (including 17 in H3, 9 in H4, 13 in H2A, 6 in H2B and 6 in H1) were modulated by minocycline. Interestingly, a much higher percentage of methylations (48 out of 83 marks) showed significant abnormal regulations in diabetes, as compared to acetylations (19 out of 52 marks). This suggests that histone methylation has a central role in the pathogenesis of diabetic retinopathy. A heatmap representing the major methylation marks, most of which showed significant differences in diabetes, are illustrated in Fig. 3. The heatmap includes 48 significantly different methylations from control to diabetic; among them, 32 methylation marks were modulated by minocycline, which highlighted the ability of minocycline in modulating the abnormal methylation levels in diabetic retinopathy.

### Alteration of histone methylations in the development of diabetic retinopathy and the effects of minocycline

**Figure 4:**
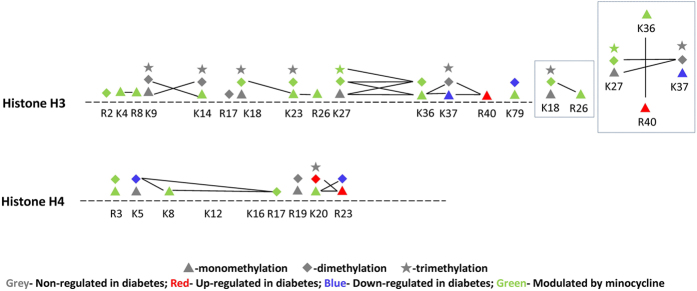
Multiple modification patterns on histone H3 and H4. Co-existing PTMs are illustrated by connecting lines. Different regulation trends were highlighted in different colors: Grey, Non-regulated in diabetes; Red, Up-regulated in diabetes; Blue, Down-regulated in diabetes; Green, abnormal regulation in diabetes was modulated by minocycline.

The histone H3 peptides aa 3–8 (TKQTAR), 9–17 (KSTGGKAPR), 18–26 (KQLATKAAR) and 27–40 (KSAPA/STGGVKKPHR), as well as the peptides of histone H4 aa 4–17 (GKGGKGLGKGGAKR) and 20–35 (KVLRDNIQGITKPAIR) were highly enriched in modifications. Table 2 and Fig. 4 display an overview of the methylations residing on histone H3 and H4, including the relative abundance of different marks quantified in this study. The complete list with average and standard deviation of all individual histone PTMs including H2A, H2B, H1 and their variantsis listed in Supplementary Table 1. For each comparison, including non-diabetic vs diabetic and diabetic vs diabetic + minocycline, we assessed statistical difference using a two tails homoscedastic t-test (significance p-value < 0.05).

**Figure 5:**
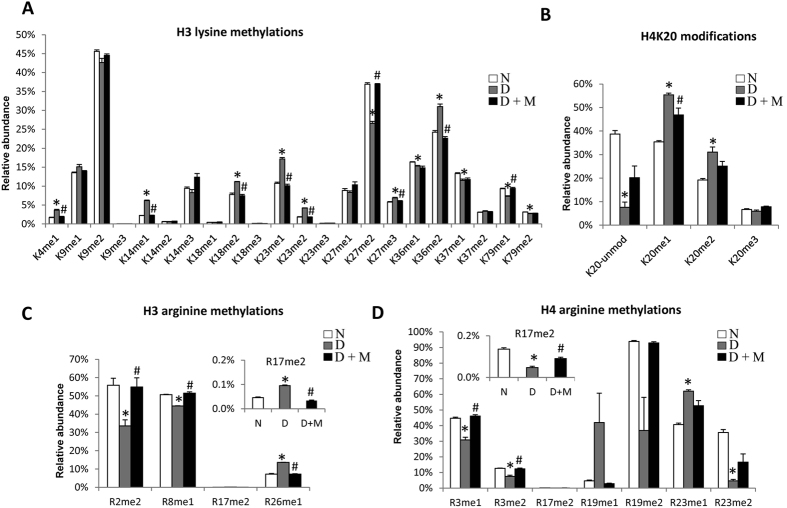
Relative abundance of PTMs on the individual methylation sites on (**A**) H3 lysine residues, (**B**) H4K20, (**C**) H3 arginine residues and (**D**) H4 arginine residues in retinas in non-diabetic rats and diabetic rats with and without minocycline treatment. Bar charts represent the relative abundance of the histone modifications. All graphs show the average of three replicates with error bars indicating SEM. Non-diabetic, diabetic with and without minocycline treatment are colored in white, black and grey, respectively.*p < 0.05 compared with non-diabetic group; ^#^p < 0.05 compared with diabetic group.

Numerous lysine residues on H3 including K4, K9, K14, K18, K23, K27, K36, K37 and K79 were methylated(Fig. 5A). Despite the low abundance of H3K4me1, a mark known to be enriched on active enhancers^19^, we could determine a significant increase in diabetes as compared to non-diabetic rat retinas. Its abundance was restored to non-diabetic levels by the minocycline treatment (non-diabetic/diabetic/diabetic + minocycline, 1.69%/3.62%/1.85%). All forms of methylations were found on H3K9, with no significant changes observed. Methylation atH3K14 was observed in lower abundances, as expected, since this site is generally mostly acetylated or unmodified. The low abundant K14me1 increased significantly in diabetes, and re-established to normal levels after minocycline treatment (2.21%/6.19%/2.23%). H3K18 was largely unmodified, but low percentages of all forms of methylations were detected. For instance, H3K18me2 was found to be significantly more abundant in diabetes, also restored by minocycline treatment (7.79%/11.17%/7.44%). H4K23me1 and H3K23me2 are examples of other marks increased in diabetes and modulated by minocycline treatment (K23me1 10.72%/17.16%/10.08%, K23me2 1.83%/4.22%/1.78%). Similar observations were made for H3K27me, H3K36me and H3K79me (Fig. 5).

Histone H4 was also decorated by methylations. In particular, all degrees of methylation were found on H4K20 (Fig. 5B), ranked in decreasing abundance as me1, me2 and me3. H4K20me1 and H4K20me2 were significantly increased in diabetes (non-diabetic/diabetic:K20me1–35.37%/55.41%;K20me2–19.24%/31.07%). Interestingly, the abnormal elevated level of H4K20me1 was significantly lowered to 46.83% after treatment of minocycline. H4K20me2 also followed the same trend as H4K20me1(diabetic + minocycline: 25.15%). However, we could not assess a statistically significant reduction upon minocycline treatment due to the variability of our observations.

Methylations were also detected on a variety of arginine residues on H3 (Fig. 5C). H3R2me2 and H3R8me1 were found in relatively higher abundance than H3R17me2 and H3R26me1, all of which were significantly altered in diabetes and recovered by minocycline. Both H3R2me2 and H3R8me1 significantly decreased in diabetes, whereas both H3R17me2 and H3R26me1 significantly increased in diabetes. Minocycline appeared to modulate the aberrant alterations of all of these arginine methylations (H3R2me2 55.85%/33.56%/54.86%, H3R8me1 50.69%/44.50%/51.46%, H3R17me2 0.05%/0.10%/0.03%, H3R26me1 7.20%/13.63%/7.19%). Methylations on arginine residues were also observed on H4 at residues R3, R17, R19 and R23. A majority of these poorly annotated H4 PTMs showed significant changes in retinas in diabetic rats and recovery effects after minocycline treatment (Fig. 5D). H4R3me1 was found in higher abundance than H4R3me2, both of which were significantly reduced in diabetes and normalized by minocycline (H4R3me1 44.69%/30.86%/46.06%, H4R3me2 12.67%/7.44%/12.26%). The mono- and di-methylations on H4R23 showed opposite significant regulations in diabetes: H4R23me1 greatly increase while H4R23me2 dramatically decreased, both of which were restored by minocycline (H4R23me1 40.63%/62.02%/52.71%, H4R23me2 35.58%/4.62%/16.63%, respectively). H4R17me2 was detected in low abundance, although we could determine that the aberrant reduced level in diabetes as compared to control was also recovered by minocycline (0.14%/0.05%/0.09%).

Collectively, we observed 48 methylation marks in H3 and H4 histone proteins, mostly concentrated on their N-terminal tails. Additionally, 35 methylations sites were observed from histone H2A, H2B and H1. 13 of those showed significant regulations in diabetes and the aberrant levels were modulated by minocycline treatment (Supplementary Fig. 2), highlighting that histone methylation levels in rat retina are widely affected in diabetes.

### A brief description of co-existing histone methylations

As described previously^18^, the bottom-up MS strategy employed in this study also allowed the characterization of nearby co-existing modifications due to the generation of ArgC cleaved peptides that contain multiple modifiable sites. The multiple PTMs detected on histones and their variants under non-diabetic, diabetic and diabetic + treatment groups are shown in Supplementary Table 2. A variety of 155 specific co-existing modification states were characterizedin this study. Nearly half of the co-existing histone modifications exhibited abnormal alterations in the retinas of diabetic rats, among them 59 co-existing modifications were modulatedby minocycline treatment (Table 1).

**Figure 6:**
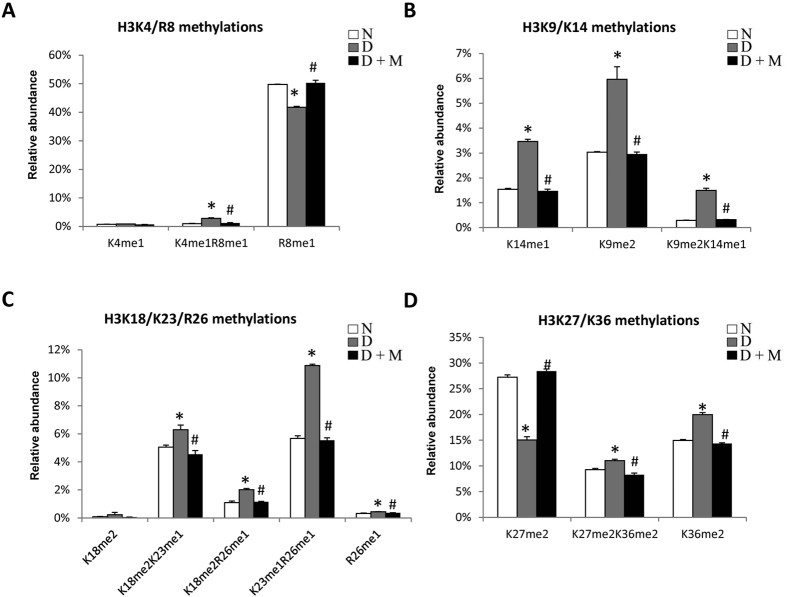
Relative abundance of co-existing methylations on histone H3 in retinas in non-diabetic rats and diabetic rats with and without minocycline treatment. MS analysis of peptides from histone H3, including (**A**) H3K4/K8methylations (aa 3–8), (**B**) H3K9/K14methylations (aa 9–17), (**C**) K18/K23/R26 methylations (aa 18–26) and (**D**) K27/K36 methylations (aa 27–40). Color white represents non-diabetic, whereas black and grey represents diabetic with and without minocycline treatment, respectively. *p < 0.05 compared with non-diabetic group; ^#^p < 0.05 compared with diabetic group.

We focused on nearby co-occurring methylations on N-terminal tails of histone H3 and H4 (Fig. 4). Co-occurring methylations such as H3K4me1R8me1, H3K9me2K14me1, H3K18me2K23me1, H3K18me2R26me1, H3K23me1R26me1, H3K27me2K36me2 and H4K20me1R23me1 significantly increased in diabetes as compared to control, and then restored by treatment with minocycline. Some of those regulations were different from the behavior of single marks. For instance, H3K4me1 was found both isolated and co-existing with R8me1 (Fig. 6A). The total amount of H3K4me1 (implying that all peptide isoforms carrying the modifications were summed to estimate the total abundance of the mark) was significantly up-regulated in diabetes and modulated by minocycline as described above (Fig. 5A). However, its isolated form remained unchanged under all conditions (Fig. 6A). The peptide containing H3R8me1 but not H3K4me was significantly down-regulated in diabetes and modulated by minocycline. However, the co-existing form H3K4me1R8me1 was found to be significantly more abundantin diabetes and lowered to normal state by minocycline (0.96%/2.78%/1.18%), suggesting a role of such co-existing methylations in disease and treatment. Similarly, no significant changes were observed for H3K9me2 when we estimated its total abundance as a single mark, while both H3 peptides (aa 9–17 KSTGGKAPR) containing H3K9me2 isolated as well as in combination with H3K14me1 (H3K9me2K14me1) increased in diabetes and minocycline was able to modulate the abnormal alterations (Fig. 6B). H3K18me2 isolated showed no alterations, however, significant increments were observed for K18me2 in the presence of H3K23me1 or H3R26me1 (Fig. 6C). Co-existing H3K23me1R26me1 in higher abundance was enriched in diabetes and was recovered by minocycline, while H3K23me1 was not observed as isolated and H3R26me1 isolated was in very low abundance. H3K27me2 isolated decreased in diabetes, however, in combination with H3K36me2, the co-existing form H3K27me2K36me2 increased in diabetes and was recovered after treatment (Fig. 6D). Collectively, we can conclude that further work is required to determine the role of combinatorial methylations and their potential cross-talk in the pathogenesis of diabetic retinopathy. However, our observations prove that combinations of histone methylations are regulated in our system, sometimes differently than the trend of single marks.

### Validations of histone methylation regulations and DNA damage

**Figure 7:**
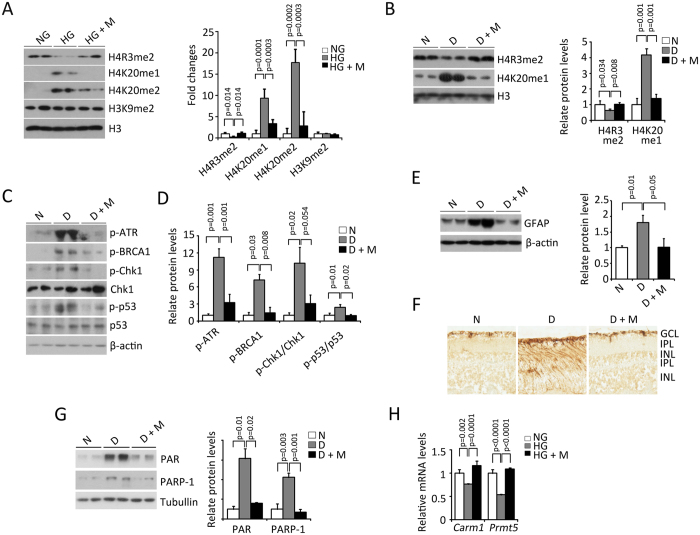
Minocycline treatment inhibited the diabetes-induced DNA damge in the retinas of diabetic rats. All blots display only two replicates, but bar plots and error bars were calculated using all replicates. (**A**). Representative western blots (left panel) with the densitometric quantitative results (right panel) of H4R3me2, H4K20me1, H4K20me2 and H3K9me2 in rMC-1 cells. (**B**) Representative western blots (left panel) with the densitometric quantitative results (right panel) of H4R3me2, H4K20me1 in the retinas. (**C**,**D**) Representative western blots (**C**) with densitometric quantitative results (**D**) of markers for DNA damage signaling in the retinas. (**E**) Representative western bolt (left panel) with the densitometric quantitative result (right panel) of GFAP in the retinas. (**F**) Representative images of GFAP staining on the retinal sections. (**G**) Representative western bolt (left panel) with the densitometric quantitative result (right panel) of PAR and PARP-1 in the retinas. (**H**) mRNA levels of *Carm1* and *Prmt5* in the rMC-1 cells. (NG: normal glucose; HG: high glucose; HG + M: high glucose with minocycline; N: Non-diabetic rats; D: Diabetic rats; D + M: Diabetic rats which treated with minocycline; individual p-values were marked).

Validation was performed using a different set of rat retinas from the samples used for MS. Specifically, we used 4, 4 and 4 rats for non-diabetic, diabetic and diabetic + minocycline conditions, respectively. Notably, only one retina per rat was used for the validation; thus, each sample corresponds to a different biological replicate. Moreover, we used rat Müller cell lines (rMC-1) to cross-validate our findings (N = 3–5 independent cultures for each group). We selected four different commercial antibodies, anti-H4R3me2, H4K20me1, H4K20me2 and H3K9me2, for western blotting. The choice was based on antibodies availability and interest in the specific PTMs. rMC-1 cells were first used to investigate whether hyperglycemia *per se* can induce histone methylation changes *in vitro*. After high glucose treatment, H4R3me2 was dramatically reduced to 26% of the control level, while H4R3me2 level was increased to 105% of the control level after treated with minocycline in high glucose condition (Fig. 7A). Meanwhile, compared to those of controls, high glucose treatment increased the H4K20me1 and H4K20me2 levels by 9.3-fold and 17.7-fold, respectively, while their levels were reduced to 3.3-fold and 2.9-fold, respectively, upon minocycline treatment (Fig. 7A). On the other hand, the levels of H3K9me2 were not significantly changed among the groups (Fig. 7A). All these results are consistent with MS data.

We further verified H4R3me2 and H4K20me1 by performing western blotting on rat retinas. In diabetic retinas, the level of H4R3me2 was reduced to 63% of that of non-diabetic retinas, while it was increased to 103% of that of non-diabeticretinas after minocycline treatment (Fig. 7B). Meanwhile, the level of H4K20me1 was increased to 4.2-fold of that of non-diabetic retinas, and it was decreased to 1.4-fold of that of non-diabetic retinas after minocycline treatment (Fig. 7B).

Increased mono- and di-methylation of H4K20 are known to be associated with DNA damage^20^,^21^. Moreover, our previous studies demonstrated that H4K20me1/me2 were significantly increased after retinal I/R^18^, as well as increased DNA damage response^18^,^22^. Thus, we verified whether the increased H4K20me1 was also associated with increased activity of the DNA damage response in the retinas of diabetic rats. Four markers of DNA damage response were examined in the retinas: phosphorylated-ATR, phosphorylated-BRCA1, phosphorylated-Chk1 and phosphorylated-p53. As compared to non-diabetic retinas, they were all significantly increased in retinas of diabetic rats (Fig. 7C,D). Furthermore, administration of minocycline significantly repressed these diabetes-induced changes in the retinas (Fig. 7C,D). We also included in the analysis of GFAP, which is a marker of glial activation, typical phenotype of diabetic retinopathy. GFAP was dramatically increased in the retinas of diabetic rats. The treatment with minocycline significantly reduced the diabetes-induced up-regulation of GFAP both by western blot analysis (Fig. 7E) and immuno-histochemical staining (Fig. 7F), indicating that minocycline suppressed the diabetes-induced glial activation.

It has been reported that minocycline prevents neuronal cell death by inhibiting the activity of PARP-1 (poly(ADP-ribose) polymerase-1)^23^, a key enzyme involved in DNA damage^24^. Previous studies by us have demonstrated that PARP-1 inhibitors prevent the retinal lesions in rodent models of diabetes and I/R injury^25^,^26^. Thus, we wonder whether the effect of minocycline on the DNA damage response is through its inhibitory effects on PARP-1 or the activity of PARP-1. Compared to that of the nondiabetic retinas, the protein level of PARP-1 was increased to 4.2-fold in the diabetic retinas and reduced to 0.7-fold after minocycline treatment. Furthermore, compared to that of the nondiabetic retinas, the activity of PARP-1 (demonstrated by protein poly(ADP-ribosyl)ation (PARylation, PAR)) was significantly increased to 6.2-fold in the diabetic retinas and reduced to 1.6-fold after minocycline treatment (Fig. 7G). Consistent with *in vivo* results, minocycline treatment also significantly reduced the high glucose-induced elevation of PAR and PARP-1 in rMC-1 cells (Supplementary Fig. 3).

To investigate how minocycline affects the H4K20 or H4R3 methylation levels, the mRNA levels of the enzymes which are responsible for the methylation levels of these two sites were examined. Upon high glucose stress, the mRNA levels of *Carm1* and *Prmt5*, the genes encoding histone H4R3 methyltransferases, CARM1 and PRMT5, respectively^27^, were significantly reduced to 80% and 57% of those of the controls, while their levels were significantly increased to 115% and 101% of the controls, respectively, after minocycline treatment (Fig. 7H). These data suggested that minocycline might regulate H4R3me2 level by controlling the transcription levels of these key histone H4R3 methyltransferases. However, compared to those of the normal glucose condition, *Suv4-20h1, Suv4-20h2* and *Phf8*, the genes encoding the key enzymes that regulate H4K20 methylation levels^28^, were not significantly changed after high glucose treatment (Supplementary Fig. 4). Interestingly, we also found that PJ-34, a specific PARP-1 inhibitor^25^, significantly increased both H4K20me1 and H4R3me2 levels in rMC-1 cells cultured under the normal glucose condition (Supplementary Fig. 5).

## Discussion

In this study, we present a detailed quantitative map of histone methylations in the retinas of diabetic rats with and without treatment with minocycline. In summary, 83 individual histone methylation marks were characterized. 58% (48 out of 83) methylation marks in all histones showed significantly increased or decreased abundances in diabetic as compared to non-diabetic rat retinas. In particular, we observed significant changes for 19 methylation sites on H3, 9 on H4, 12 on H2A, 2 on H2B and 6 on H1 in diabetes. Interestingly, a total of 32 methylation marks exhibited recovery effect of minocycline, including 14 marks on histone H3, 5 on H4, 7 on H2A, 2 on H2B and 4 on H1 (Supplementary Table 1). Our findings emphasized that histone methylation might play a critical role in the etiology and clinical therapy of diabetic retinopathy.

In addition to single histone PTMs, our analytical strategy also allowed characterization of numerous nearby co-occurring PTMs including co-existing methylations. Half of the co-existing PTMs patterns showed abnormal regulation in diabetes, and 38% were normalized by minocycline (Supplementary Table 2). It would be valuable to employ top/middle-down proteomics^29^,^30^,^31^, which would provide a more complete picture of distant combinatorial histone PTMs.

As listed in Table 2, we compared ourcurrent study with our previousstudy on alteration of individual histone marks after retinalI/R injury in rats^18^, and found that several changeswere consistent with our findings in I/R injury, such as the relative abundance of H4K20me1, H4K20me2, H3K36me1, H3K36me2, and H3.3K37me1 in the retinas of STZ-induced diabetes. The abnormal levels of H4K20me1/me2 and H4K36me2 in diabetes recovered by minocycline are particularly interesting, because chronic retinal diseases and acute retinal diseases, such as diabetic retinopathy and I/R injury, share similar pathological lesions, including capillary degeneration, neuronal loss and glial activation^26^. Identifying the similar changes in both models might give us the hints which are the leading causes of these pathological changes in the retinas.

Alterations of histone methylation levels have been demonstrated in the retinas of diabetic rodents. After been diabetic for 4–6 months, increased H4K20me3 and reduced H3K4me1/H3K4me2 levels have been found on the promoter of *Sod2* (encodes MnSOD, a critical enzyme involved in oxidative stress defense system) in the diabetic retinas. This led to reduced *Sod2* level and increased mitochondrial damage which eventually promote the development of diabetic retinopathy^3^. More importantly, these epigenetic changes on the promoter of *Sod2* could continue for several months in the diabetic retinas even after their blood glucose levels were normalized by extensively insulin treatment^32^, indicating metabolic memory can be recorded in these histone methylation markers. Decreased H3K9me2 level on the promoter of *MMP-9* has also been found in the retinas of rats which have been diabetic for 6 months^32^. Increased PRMT4, a histone methyltransferase which is responsible for the methylation of H3R17, has been found in the retinal pigment epithelial layer of rats which have been diabetic for 2.5 months to promote cell death^33^. Although the vascular complications of diabetic retinopathy in rodents usually need 6–8 months to develop, the development of diabetes-induced glial activation usually takes less time (2–3 months)^34^. The changes of histone methylation levels can be detected as early as 2 or 3 months of diabetes, however, which histone methylation(s) is (are) the key epigenetic marker(s) responsible for the development of diabetic retinopathy is still demanding further investigation.

The increased levels H4K20me1/me2 were found not only in the retinas of diabetic rats, but also in the high glucose treated rMC-1 cells (Fig. 7A,B). However, among all the known enzymes that regulates H4K20 methylation levels, the mRNA levels of *Suv4-20h1, Suv4-20h2* and *Phf8* were not changed even after high glucose stress in cultured Müller cells (Supplementary Fig. 4), indicating that the protein level or the enzyme activity of these histone methylase or demethylase may be responsible for the dramatic elevation of H4K20me1/me2 levels upon hyperglycemic stress. Actually, increased SUV420H2 has been found in the retinas of diabetic rats^35^. Furthermore, it is also possible that an unknown histone H4K20 methytransferase or demethylase exists which is responsible for the significant changes of H4K20me1/me2 levels in the retinas or cultured retinal cells upon diabetes or hyperglycemic stress. Meanwhile, we demonstrated that the transcriptional levels of histone methytransferases which regulate H4R3 methylation were altered in hyperglycemic stress and recovered by minocycline treatment (Fig. 7H), indicating that the effects of minocycline on regulating histone methylation levels can partially explained by its ability to regulate some histone methyltransferases or de-methylases. The exact mechanisms underlying the effects of minocycline on histone methylation are more complicated than we thought, and more research needs to be done in the future.

The DNA damage signaling pathway, known to be associated with H4K20me1/me2 was also up-regulated in the retinas of diabetic rats (Fig. 7C). A similar effect was observed in the retinal I/R injury model in our previous study^18^. Furthermore, administration of minocycline significantly reduced markers of DNA damage response in the diabetic retinas (Fig. 7C). Minocycline also reduced the protein level and the activity of PARP-1 in the retinas of diabetic rats (Fig. 7G). Since PARP-1 is a key enzyme involved in DNA damage, and inhibition of PARP-1 activation by specific inhibitors prevents the retinal lesions in rodent models of diabetes and I/R injury^25^,^26^, the beneficial effect of minocycline on DNA damage might be due to its ability on inhibition of PARP-1 activation.

Besides the DNA damage, glial activation may be another pathway contributing to the development of diabetic retinopathy^34^. It has been reported thatminocycline can efficiently reduce the GFAP expression both in brain and cultured retina cells^2^,^11^. Bosco *et al*. reported thatminocycline treatment did not affect Müller gliosis in the DBA/2J mouse, a chronic glaucoma mouse model^36^. The discrepancy between their results and the present study may due to (1) different disease model used (a chronic glaucoma model vs. a diabetic retinopathy model); and (2) different ways of minocycline administration (gavage vs. intraperitoneal injection).

In conclusion, our results show that methylations are highly altered in the development of diabetic retinopathy in rodent model, and minocycline treatment has a dramatic impact on such PTMs, reverting about 67% to non-diabetic levels. As a vital epigenetic modification, alteration of histone methylation is clearly associated with the development diabetic retinopathy in rodents, and H4K20me1/me2-associated DNA damage pathway might be the therapeutic targets for this ocular disease.

## Methods

### Materials

Pure water was obtained from a Milli-Q system (Millipore, Bedford, MA). ArgC was from G-Biosciences (USA). Poros Oligo R3 reversed-phase material was from PerSeptive Biosystems (Framingham, MA). All other reagents and solvents were of the highest commercial quality and were used without further purification.

### Rat model of diabetes and treatment with minocycline

Sprague-Dawley rats were obtained from the ABSL-III laboratory of Wuhan University (China). Diabetes was induced as we previously described^2^. The total experiment involved 24 rats. One week after streptozocin (STZ) (Amresco, Solon, OH) injection, 17 rats with non-fasted blood glucose (NFBG) above 250 mg/dl (measured by OneTouch blood glucose meter, LifeScan, Milpitas, CA) were considered as diabetic. One week after being diagnosed as diabetic, 9 rats were treated with minocycline (25 mg/kg body weight) by intraperitoneal injection 5 days/week. Eight weeks after administration of minocycline, rats were sacrificed. The duration of diabetes chosen in the present study is because a lot of biochemical changes which contribute to the development of diabetic retinopathy have been reported to happen in the rats after 2 months of diabetes^35^. All procedures involving the animal study were approved by the Committee on Ethics in the Care and Use of Laboratory Animals of College of Life Sciences at Wuhan University. All methods were performed in accordance with the relevant guidelines and regulations.

### Cell culture

rMC-1 cells were cultured in 5 mM glucose Dulbecco’s Modified Eagle Medium (DMEM) media (Hyclone, South Logan, UT) supplementedwith 10% fetal bovine serum (FBS) (Hyclone) and 1% sodium pyruvate (Hyclone). Cells were incubated at 37 °C in 5% CO_2_. When cells reached 80% confluence, some of the media were replaced with 25 mM glucose DMEM media with or without 30 μM minocycline (Sigma, Steinheim, Germany), while other cells were kept in 5 mM glucose DMEM media as controls. Cells were cultured under different glucose media for 48 hours until harvested. To inhibit the PARP-1 activity, 90–100% confluent rMC-1 cells were treated with or without 1 μm PJ-34 for 24 hours in 5 mM glucose DMEM media.

### Quantitative real-time PCR (qPCR)

RNA was extracted from cells using RNAiso Plus (Takara Biotechnology, 9109) as previously described^37^. cDNA synthesis was performed using the M-MLV First Strand Kit (Invitrogen, 28025-021). Primer sequences for the target genes are provided in Supplementary Table 5. qPCR were performed as previously described^38^. The formation of a single product for each primer set was confirmed by observing only one peak in the melting curve for each reaction. *Actb* were used as internal controls. The relative difference is expressed as the fold change calculated using the 2^−ΔΔCT^ method.

### Histone Extraction

Histones were extracted as we previously described^18^. Briefly, freshly isolated retinas were homogenized in hypotonic lysis buffer with PhosSTOP (Roche, Basel, Switzerland) and Protease Inhibitor Cocktail (Roche). The samples were further lysed by hypotonic lysis buffer with 0.1% NP-40 for 10 min on ice. The nuclei were collected and histones were extracted by incubating the nuclei with 0.2 M H_2_SO_4_ for 2 hours at 4 °C. Histones were then precipitated using 33% trichloroacetic acid (TCA) for 1 hour at 4 °C. After being washed with acetone, histones were dissolved in dH_2_O, and the concentration was estimated using the Bio-Rad Protein Assay (Bio-Rad, Hercules, CA, USA). Histones were extracted from non-diabetic rats (n = 3), diabetic rats (n = 4) and diabetic rats treated with minocycline (n = 5), individually, and samples within each group were combined to ensure sensitivity for the MS analysis.

### Histone digestion and nanoLC-MS

Precipitated histones were resuspended in 100 mM NH_4_HCO_3_, 1 mM calcium acetate and 7.5 mM DTT. Samples were digested using ArgC at an enzyme:sample ratio of 1:50 overnight at 37 °C. Digestion was interrupted by adding 1% TFA to the solution. Samples were loaded and separated with an EASY-nLC nanoHPLC (Thermo Scientific, San Jose, CA). nanoLC was set up with a 75 μm ID × 17 cm Reprosil-Pur C18-AQ (3 μm; Dr. Maisch GmbH) nano-column. Injection volume was set to 5 μL. The HPLC gradient was 0–34% solvent B (A = 0.1% formic acid; B = 95% MeCN, 0.1% formic acid) over 60 minutes and from 34% to 100% solvent B in 5 minutes at a flow-rate of 300 nL/min. Liquid chromatography (LC) was coupled with an LTQ-Orbitrap Velos Pro mass spectrometer (Thermo Scientific, San Jose, CA). Nanoelectrospray (Proxeon) was used with a spray voltage of 2.3 kV. No sheath, sweep, and auxiliary gasses were used, and capillary temperature was set to 275 °C. The instrument was used in data-dependent MS/MS acquisition (DDA) mode^39^. Acquisition was performed in the Orbitrap for MS with a resolution of 60,000 (full-width at half-height of 400 *m*/*z*) and in the linear ion trap (LTQ) for MS/MS. MS *m*/*z* acquisition window was set at 290–1400. Precursor charge states 1+ and unassigned were excluded. The 10 most intense ions over a threshold of 5,000 counts were isolated for fragmentation in the LTQ using CID, with an activation Q value of 0.25 and activation time of 15 ms. The dynamic exclusion time was set to 60 sec.

### Data processing and database searching

Data were processed using Proteome Discoverer v1.3.0.339 (Thermo Scientific). Mascot v2.3 (Matrix Science) was used for database searching. A list of rat and mouse histones was downloaded from Uniprot (updated at 5/09/2011). Arg-C was selected as digestion enzyme and two missed cleavages were allowed. MS mass tolerance was set to 8 ppm, while MS/MS tolerance was set to 0.6 Da. No fixed modifications were allowed, while dynamic modifications included were acetylation (protein N-term and K), methylation (KR), dimethylation (KR) and trimethylation (K). Peptide validation was performed using Fixed Value Peptide validator, which uses a fixed score threshold to estimate peptide confidence without performing decoy database searching (this is inapplicable when a large number of dynamic modifications is included). Peptides were filtered with the following parameters: mascot score >20, peptide rank: 1 and peptide false discovery rate <5%. The quality of MS/MS spectra was manually evaluated to ensure the confidence threshold was set correctly. Quantification was performed using Progenesis LC-MS v4.1 (Nonlinear dynamics) using default settings for peak area extraction. Each quantified peptide was divided by the quantification of the respective peptide in all the modified states (including unmodified form) in order to obtain an estimation of the modified sites in percentage of the total signal obtained for a particular histone species. All quantitative results were expressed as the mean ± SD (standard deviation). For two-group comparison, data was analyzed by homoscedastic t-test (two samples equal variance) based on three technical replicates. Differences were considered statistically significant when the p-values were less than 0.05.

### Immuno-histochemical staining for GFAP

Retinal sections were deparaffinized and rehydrated as we previously reported^40^. For immuno-histochemical staining, retinal sections were incubated overnight with a rabbit anti-histone GFAP antibody (1:2000 dilution, Abcam, Cambridge, UK). After extensive washing, sections were further incubated with a biotinylated anti-rabbit antibody (Vector Laboratories, Burlingame, CA). Positive staining was visualized by DAB substrate reaction (Vector laboratories) following the ABC kit protocol (Vector laboratories). High resolution pictures were taken with an Olympus BX60 microscope.

### Western blots and Statistical analysis

A different set of eye retinas was used for western blotting. Specifically, we isolated retinas and cells, and sonicated them in ice-cold RIPA buffer separately for 4 non-diabetic, 4 diabetic and 4 diabetic treated with minocycline rats. As note, only one eye per rat was used for this experiment. Protein concentration was estimated as previously reported^18^. 20 μg proteins were separated by SDS-PAGE, and electroblotted onto PVDF membrane(Millipore, Billerica, MA, USA). Antibodies for H3K9me2 (1:5000), H3 (1:10000), β-actin (1:20000), GFAP (1:100000) and Chk1 (1:5000) was obtained from Abcam(Cambridge, England); antibodies for H4K20me1 (1:10000) andH4R3me2 (1:10000) were obtained from Active Motif (Carlsbad, CA, USA); antibodies for phosphorylated-ATR (Ser428) (1:1000), phosphorylated-BRCA1 (Ser1524) (1:1000), phosphorylated-Chk1(Ser345) (1:1000) and phosphorylated-p53(Ser15) (1:1000) were obtained from Cell Signaling Technology(Beverly, MA, USA); antibody for p53 (1:500) was obtained from Santa Cruz (Santa Cruz, CA, USA). Protein bands detected by the antibodies were visualized by enhanced chemiluminescence (ECL, Pierce, IL, USA) and evaluated by the Quantity One 1-D Analysis Software (Bio-Rad). The levels of modified histones were first estimated relatively to the total histone H3 present in the same sample. Quantification of the other target proteins were compared to β-actin in the same sample, and their relative expression level across conditions was normalized by the control rats (non-diabetic). All results were expressed as the mean ± SD. Differences were considered statistically significant when the p-value was lower than 0.05 using a two-tailed homoscedastic t-test.

## Additional Information

**How to cite this article:** Wang, W. *et al*. Abnormal levels of histone methylation in the retinas of diabetic rats are reversed by minocycline treatment. *Sci. Rep.* **7**, 45103; doi: 10.1038/srep45103 (2017).

**Publisher's note:** Springer Nature remains neutral with regard to jurisdictional claims in published maps and institutional affiliations.

## Supplementary Material

Supplementary Information

Supplementary Tables

## Figures and Tables

**Table 1:** Summary of identified and quantified single and co-existing histone PTMs in the present study.

	Identified single marks	Down-regulated in diabetes	Modulated by minocycline	Up-regulated in diabetes	Modulated by minocycline
H1	14	3	2	5	4
H2A	37	6	4	12	9
H2B	22	3	3	3	3
H3	43	11	7	11	10
H4	19	9	7	4	2
	**Identified co-existing marks**	**Down-regulated in diabetes**	**Modulated by minocycline**	**Up-regulated in diabetes**	**Modulated by minocycline**
H1	14	4	3	5	3
H2A	27	4	2	6	4
H2B	14	2	2	2	2
H3	63	12	7	22	21
H4	37	13	10	6	5

**Table 2:** Summary of the individual PTMs regulations under three conditions on histone H3 and H4.

Group	PTMs	N	D	D + M	In D	In D + M	In I/R
H3	K4me1	1.7 ± 0.2%	3.6 ± 0.4%	1.8 ± 0.3%	↑	+	
H3	K14me1	2.2 ± 0%	6.2 ± 0.2%	2.2 ± 0.2%	↑	+	N/A
H3	R17me2	0.05 ± 0.01%	0.1 ± 0.01%	0.03 ± 0%	↑	+	N/A
H3	K18me2	7.8 ± 0.7%	11.2 ± 0.1%	7.4 ± 0.6%	↑	+	N/A
H3	K23me1	10.7 ± 0.6%	17.2 ± 0.7%	10.1 ± 0.8%	↑	+	N/A
H3	K23me2	1.8 ± 0.1%	4.2 ± 0%	1.8 ± 0.1%	↑	+	N/A
H3	R26me1	7.2 ± 0.6%	13.6 ± 0.1%	7.2 ± 0.4%	↑	+	↓
H3	K27me3	5.8 ± 0.2%	6.9 ± 0.3%	6 ± 0.2%	↑	+	
H3	K36me2	24.2 ± 0.8%	31 ± 1.2%	22.6 ± 0.7%	↑	+	↑
H3	R2me2	55.9 ± 6.6%	33.6 ± 5.7%	54.9 ± 8.7%	↓	+	N/A
H3	R8me1	50.7 ± 0.3%	44.5 ± 0.2%	51.5 ± 1.3%	↓	+	N/A
H3	K27me2	36.9 ± 0.7%	26.6 ± 0.9%	37 ± 0.2%	↓	+	
H3	K79me1	9.3 ± 0.3%	7.3 ± 0.2%	9.4 ± 0.5%	↓	+	
H3.3	K36me1	13 ± 0.5%	8.2 ± 2.3%	13.5 ± 2.1%	↓	+	N/A
H3.3	R40me1	0.02 ± 0.01%	0.2 ± 0.1%	0.5 ± 0.2%	↑		
H3	K36me1	16.3 ± 0.2%	15.3 ± 0.3%	14.8 ± 0.8%	↓		↓
H3	K37me1	13.3 ± 0.4%	11.5 ± 0.6%	11.7 ± 0.7%	↓		N/A
H3	K79me2	3.1 ± 0.1%	2.7 ± 0.1%	2.7 ± 0.2%	↓		
H3.3	K37me1	15.1 ± 0.8%	9.3 ± 2.5%	14.9 ± 2.5%	↓		↓
H3	K9me1	13.5 ± 0.3%	15.1 ± 1.1%	14 ± 0.3%			
H3	K9me2	45.6 ± 0.6%	42.6 ± 1.9%	44.5 ± 0.7%			
H3	K9me3	0.01 ± 0%	0.02 ± 0.01%	0.03 ± 0%			
H3	K14me2	0.6 ± 0.1%	0.6 ± 0.1%	0.7 ± 0%			N/A
H3	K14me3	9.4 ± 0.6%	8.3 ± 1.5%	12.4 ± 1.6%			N/A
H3	K18me1	0.4 ± 0%	0.4 ± 0.1%	0.5 ± 0.1%			
H3	K18me3	0.1 ± 0%	0.2 ± 0%	0.1 ± 0%			
H3	K23me3	0.2 ± 0%	0.2 ± 0%	0.2 ± 0%			
H3	K27me1	8.9 ± 0.9%	8.3 ± 0.7%	10.3 ± 1.4%			
H3	K37me2	3.1 ± 0.1%	3.4 ± 0.3%	3.2 ± 0.2%			N/A
H3.3	K27me1	3.3 ± 1.3%	8.7 ± 8.1%	1.9 ± 0.8%			
H3.3	K27me2	30.2 ± 1%	35.4 ± 4.8%	32 ± 3.2%			
H3.3	K27me3	34.1 ± 2.3%	30.3 ± 5.8%	30.6 ± 1.6%			N/A
H3.3	K36me2	80.9 ± 0.4%	80.5 ± 5.3%	81.5 ± 3.2%			
H3.3	K37me2	3.3 ± 1.3%	8.7 ± 8.1%	1.9 ± 0.8%			
H3.3	K37me3	0.05 ± 0.01%	0.04 ± 0.01%	0.03 ± 0.02%			N/A
H4	K20me1	35.4 ± 0.9%	55.4 ± 1.2%	46.8 ± 5.1%	↑	+	↑
H4	R3me1	44.7 ± 1.2%	30.9 ± 2.9%	46.1 ± 1.5%	↓	+	
H4	R3me2	12.7 ± 0.3%	7.4 ± 1.4%	12.3 ± 0.9%	↓	+	
H4	K8me1	0.1 ± 0%	0 ± 0%	0.1 ± 0%	↓	+	
H4	R17me2	0.1 ± 0%	0 ± 0%	0.1 ± 0%	↓	+	N/A
H4	K20me2	19.2 ± 1%	31.1 ± 3.8%	25.2 ± 3.4%	↑		↑
H4	R23me1	40.6 ± 1.7%	62 ± 1.5%	52.7 ± 5.7%	↑		↓
H4	K5me2	1.3 ± 0.1%	1.1 ± 0.02%	1.3 ± 0.2%	↓		N/A
H4	R23me2	35.6 ± 3.2%	4.6 ± 1.7%	16.6 ± 9.1%	↓		
H4	K5me1	1.9 ± 0.1%	1.7 ± 0.6%	1.5 ± 0.1%			
H4	R19me1	4.6 ± 0.9%	41.9 ± 32.6%	2.8 ± 0.7%			
H4	R19me2	93.9 ± 1.1%	36.9 ± 36.6%	92.9 ± 1.3%			N/A
H4	K20me3	6.7 ± 0.6%	5.9 ± 0.9%	7.8 ± 0.5%			

The site, type and relative abundance of each PTM is indicated. ↑/↓represent histone marks significantly up-/down-regulatedin diabetic retinopathy, calculated by a two-tailedhomoscedastic t-test with a p-value < 0.05 based on three technical replicates. Those which showed abnormal regulation in diabetic retinopathy and were modulated by the minocycline treatment were marked with + . The regulated histone marks in our previous work inacute ischemia and reperfusion (I/R) injury were also marked with ↑/↓. N: Non-diabetic; D: Diabetic;D + M: Diabetic + Minocycline. N/A represents not found in our previous study.
