# Supplementary material for: Abnormal levels of histone methylation in the retinas of diabetic rats are reversed by minocycline treatment
Source: Sci Rep. 2017 Mar 24;7:45103. doi: 10.1038/srep45103 (PMC5364468; doi:10.1038/srep45103)
Supplement: Supplementary Information [file srep45103-s1.pdf]

Supporting information

Abnormal levels of histone methylation in the retinas of diabetic rats are reversed by minocycline treatment

Wenjun Wang [1,#], Simone Sidoli [2,$,#], Wenquan Zhang[1], Qing Wang[1], Leilei Wang [1], Ole Nørregaard Jensen[2], Lin Guo[1], Xiaolu Zhao[1,*], Ling Zheng[1,*]

[1]Hubei Key Laboratory of Cell Homeostasis, College of Life Sciences, Wuhan University, Wuhan, P.R.China, 430072

[2]Department of Biochemistry and Molecular Biology and VILLUM Center for Bioanalytical Sciences, University of Southern Denmark, DK-5230 Odense M, Denmark.

[*]Corresponding authors:

Ling Zheng, Professor

College of Life Sciences, Wuhan University, Wuhan, P.R.China, 430072

Email lzheng@whu.edu.cn Tel. + 86-27-68755559 Fax + 86-27-68755559

Xiaolu Zhao, Associate Professor

College of Life Sciences, Wuhan University, Wuhan, P.R.China, 430072

Email zhaoxiaolu@whu.edu.cn Tel. + 86-27-68753889 Fax + 86-27-68753797

[#]These authors contributed equally to this work.

[$]Current address: Epigenetics Program, Department of Biochemistry and Biophysics, Perelman School of Medicine, University of Pennsylvania, United States, PA-19104

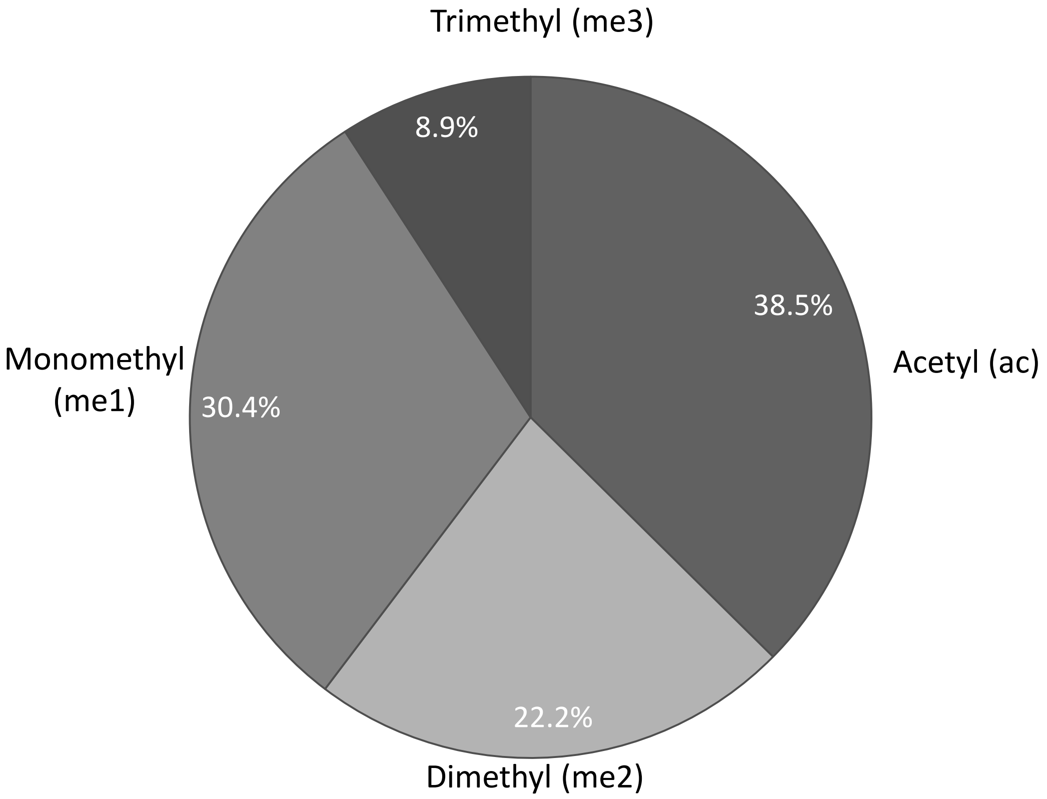

Supplementary Fig. 1: Relative abundance of the quantified PTMs on all detected histones.

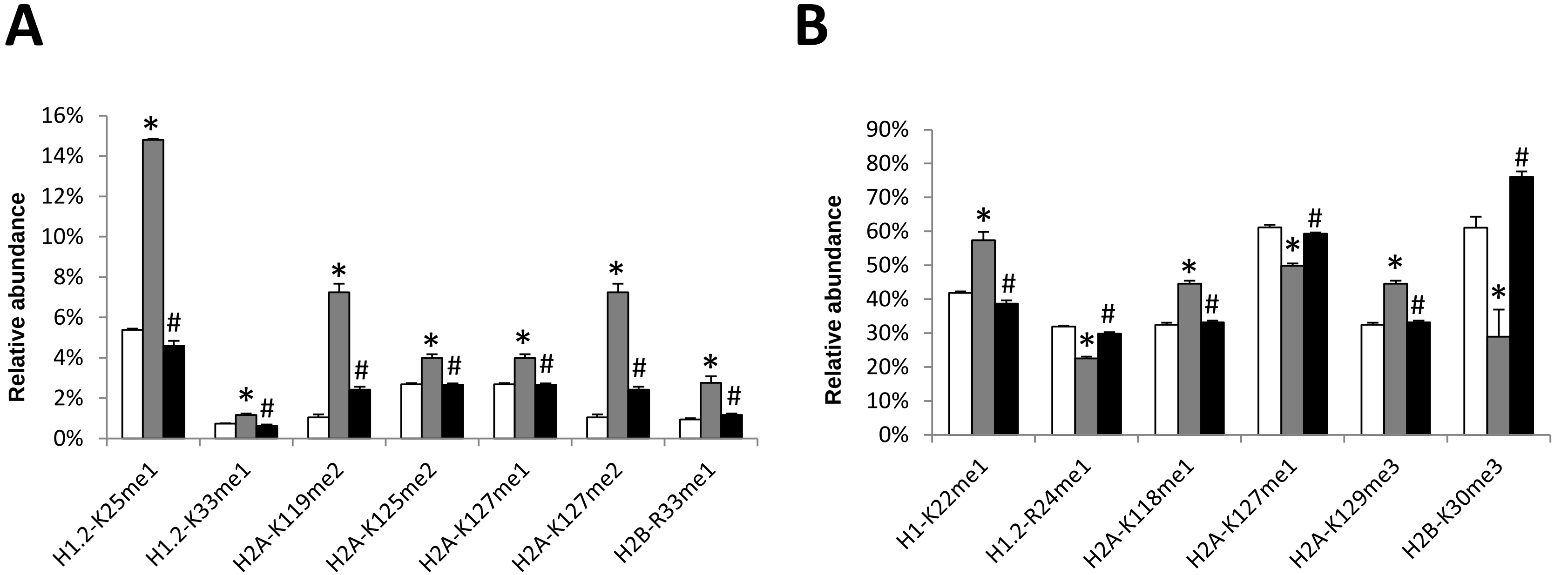

Supplementary Fig. 2: Relative abundance of PTMs on the methylation sites on H1, H2A and H2B. Color white represents control, whereas grey and black represents diabetes without and with minocycline treatment, respectively.

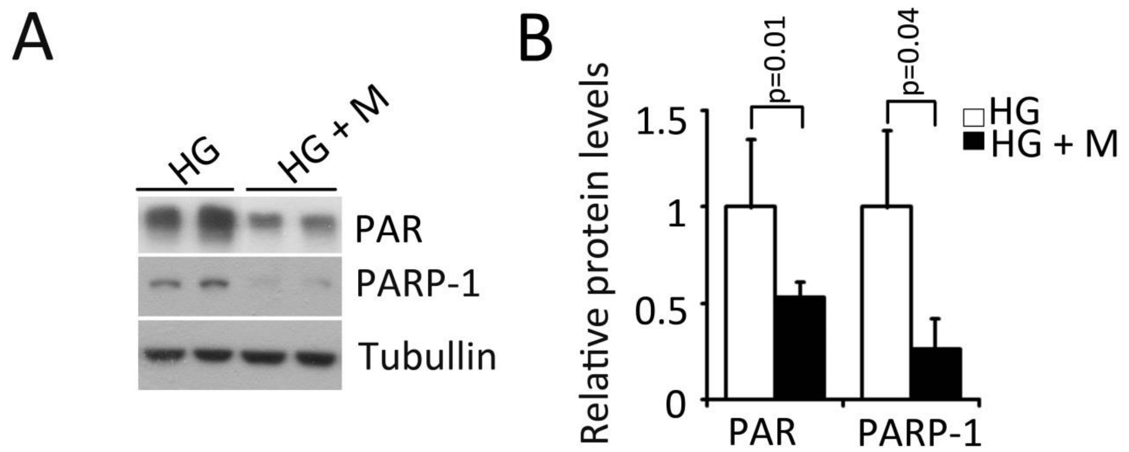

Supplementary Fig. 3: (A-B) Representative western bolt (A) with the densitometric quantitative result (B) of PAR and PARP-1 in rMC-1. (N= 3-6; HG: high glucose; HG +M: high glucose with minocycline)

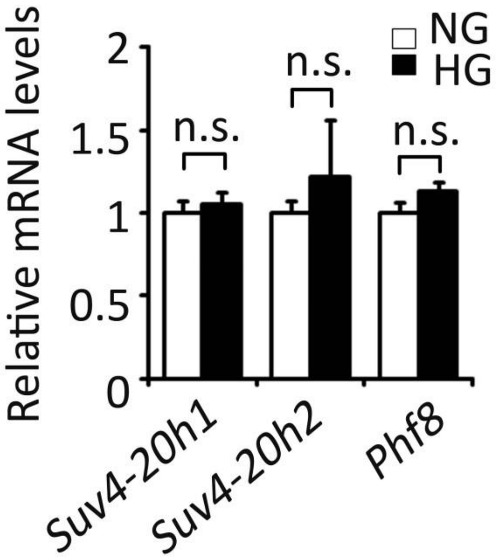

Supplementary Fig. 4: mRNA levels of the indicated genes after high glucose treatment. (n.s. : no significant).

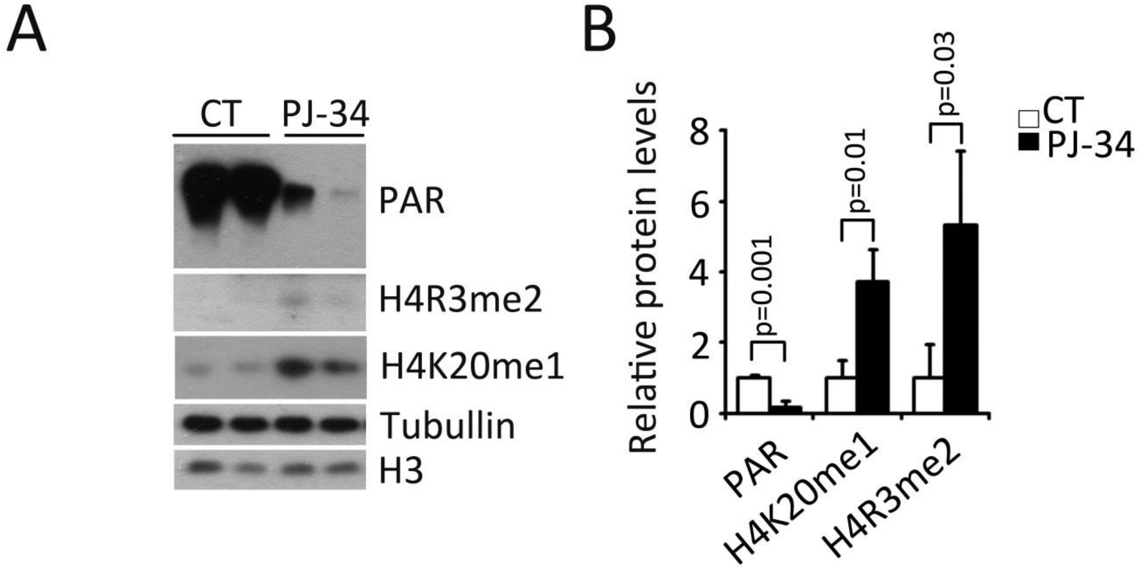

Supplementary Fig. 5: (A-B) Representative western bolt (A) with the densitometric quantitative result (B) of PAR, H4R3me2 and H4K20me1 in rMC-1 after the PJ-34 treatment. (N= 3; PJ-34: inhibitor of PARP-1).

Supplementary Table 1: Relative abundances of single histone marks.

Supplementary Table 2: Relative abundances of co-existing histone marks.

Supplementary Table 3: Relative abundances of all identified histone peptides by LC-MS/MS.

Supplementary Table 4: Information of all identified peptides by LC-MS/MS.
